# Too Many Meals

**Published:** 1891-02

**Authors:** 


### TOO MANY MEALS.

There is, says Sir Andrew Clark, growing up in the nursing world a habit of having far too frequent meals in the day, and far too much variety at them. Meals should not be multiplied in the way they now are. The plan of having tea before breakfast, lunch at eleven o'clock, then dinner, tea again in the afternoon, and supper at night is about the worst plan that can be adopted. Nurses should have three meals a day, good sensible meals of fresh, simple, nourishing food. Women have but one notion about food, and that is that the oftener they eat and the more varied the food the better they will be. This is a mistake. Having three simple meals of plain,nourishing food,and nothing between them, is the best way of sustaining the health and of supporting the body. Above all, tinned meat should be avoided, as they form a most dangerous kind of food. The diet of twenty-five years ago, when nurses had plain bread and butter for breakfast at half-past six, a dinner consisting of a joint at one, and a cold meat supper, was much more fitting for the maintenance of health and the support of the working powers than the diet of the present day.

Children should be well fed. Some are overfed, but far more are underfed. They should be fed on abundance of bread, milk, eggs, rice, barley, and oatmeal in every form. As a rule, a child should be allowed to eat as much as he will of plain, nourishing food. Parents have some very curious notions on the subject of eating. Children often have a hatred, and sometimes even a horror, of certain articles of food. Fat, underdone meat, eggs, pork, liver, and other things are often hated by children. In such cases it is unwise to press them. Food eaten with aversion or under threats is pretty sure to disagree ; and often a child really knows far better what is suited for him than the obstinate parent. Three good meals a day are best for children.

